# The identification and differential expression of *Eucalyptus grandis* pathogenesis-related genes in response to salicylic acid and methyl jasmonate

**DOI:** 10.3389/fpls.2013.00043

**Published:** 2013-03-06

**Authors:** Ronishree Naidoo, Linda Ferreira, Dave K. Berger, Alexander A. Myburg, Sanushka Naidoo

**Affiliations:** ^1^Department of Genetics, University of PretoriaPretoria, South Africa; ^2^Forestry and Agricultural Biotechnology Institute, University of PretoriaPretoria, South Africa; ^3^Department of Plant Science, University of PretoriaPretoria, South Africa

**Keywords:** *Eucalyptus*, salicylic acid, methyl jasmonate, PR genes, defence, *PR2*, *PR4*

## Abstract

Two important role players in plant defence response are the phytohormones salicylic acid (SA) and jasmonic acid (JA); both of which have been well described in model species such as *Arabidopsis thaliana*. Several pathogenesis related (PR) genes have previously been used as indicators of the onset of SA and JA signaling in *Arabidopsis*. This information is lacking in tree genera such as *Eucalyptus*. The aim of this study was to characterize the transcriptional response of PR genes (*EgrPR2*, *EgrPR3*, *EgrPR4*, *EgrPR5*, *and EgrLOX*) identified in *Eucalyptus grandis* to SA and methyl jasmonate (MeJA) treatment as well as to qualify them as diagnostic for the two signaling pathways. Using the genome sequence of *E. grandis*, we identified candidate *Eucalyptus* orthologs *EgrPR2*, *EgrPR3*, *EgrPR4*, *EgrPR5*, *and EgrLOX* based on a co-phylogenetic approach. The expression of these genes was investigated after various doses of SA and MeJA (a derivative of JA) treatment as well as at various time points. The transcript levels of *EgrPR2* were decreased in response to high concentrations of MeJA whereas the expression of *EgrPR3* and *EgrLOX* declined as the concentrations of SA treatment increased, suggesting an antagonistic relationship between SA and MeJA. Our results support *EgrPR2* as potentially diagnostic for SA and *EgrPR3*, *EgrPR4*, *and EgrLOX* as indicators of MeJA signaling. To further validate the diagnostic potential of the PR genes we challenged *E. grandis* clones with the fungal necrotrophic pathogen *Chrysoporthe austroafricana*. The tolerant clone showed high induction of *EgrPR2* and decreased transcript abundance of *EgrPR4*. Pre-treatment of the susceptible genotype with 5 mM SA resulted in lesion lengths comparable to the tolerant genotype after artificial inoculation with *C. austroafricana.* Thus expression profiling of *EgrPR2* and *EgrPR4* genes could serve as a useful diagnostic approach to determine which of the two signaling pathways are activated against various pathogens in *Eucalyptus*.

## Introduction

The defence mechanisms that are employed by plants to deter pathogens have been well-studied in various model organisms such as *Arabidopsis thaliana*. These model systems have created a foundation for understanding general host responses to pathogens. Following the plants perception of an invading pathogen, a plethora of defences responses are activated. Among these responses is the activation of various phytohormone signaling molecules including salicylic acid (SA), jasmonic acid (JA), ethylene (ET), abscisic acid (ABA), auxin, cytokinins (CK), gibberelins (GA), and brassinosteriods (BR). In particular, the phytohormones SA and JA have been extensively investigated in various pathosystems. These studies have shown that biotrophic pathogens are impeded by the activation of the SA pathway whereas necrotrophic pathogens are targeted by induction of JA and ET signaling pathways (Glazebrook, 2005). Each of these signaling cascades has been shown to involve the activation of certain signature defence genes, e.g., Pathogenesis Related (PR) genes, which can be representative of the induction of a pathway (Reymond and Farmer, 1998).

Stimulation of the SA pathway can be represented by an increase in the expression levels of *PR1*, *PR2*, and *PR5* defence genes (Kunkel and Brooks, 2002; Delaure et al., 2008). *Arabidopsis* SA signaling mutants *npr1*, *nim1*, and *sai1* as well as plants expressing the bacterial salicylate hydroxylase (*nahG*) are all impaired in their ability to induce expression of the *PR1*, *PR2*, and *PR5* thereby indicating that these PR candidates can be used as a measure of SA signaling induction (Cao et al., 1994; Delaney et al., 1995; Shah et al., 1997). In the case of *eds* and *pad* mutants, there is a lack of SA signaling thereby allowing for increase in JA signaling due to the lack of antagonism by SA (Zhou et al., 1998; Gupta et al., 2000; Nawrath et al., 2002; Glazebrook et al., 2003). Transgenic plants over-expressing these SA signature defence genes have also been shown to result in increased resistance against pathogens such as *Phytophthora parasitica* and *Alternaria alternata* (Alexander et al., 1993; Jach et al., 1995). Induction of a derivative of JA, MeJA, can be represented in *Arabidopsis* by an increase in the expression levels of *PR3*, *PR4*, Vegetative Storage Protein (*VSP*), and Lipoxygenase (*LOX*). Over-expression of these proteins has also been shown to confer resistance to *Phytophthora nicotianae* and *Rhizoctonia solani* (Boter et al., 2004; Mishina and Zeier, 2007; Kusajima et al., 2010). Mutants of the JA signaling pathway in *Arabidopsis*, e.g., *fad3/7/8*, *coi1*, *and jar1* have been shown to inhibit the expression of *PR3*, *PR4*, *VSP*, and *LOX* and thus increase the susceptibility of the mutant lines to numerous pathogens (Staswick et al., 1998; Vijayan et al., 1998; Norman-Setterblad et al., 2000). Additional JA mutants, *mpk4* and *ssi2*, display increased levels of *PR1*, *PR2*, and *PR5* whilst impaired in JA defence gene expression, thereby indicating that these mutants are involved in JA and SA antagonism (Petersen et al., 2000; Kachroo et al., 2001; Shah et al., 2001). Consequently *PR3*, *PR4*, and *LOX* defence genes can be used as indicators for the onset of JA signaling. One can thus refer to *PR2* and *PR5* as signature defence response genes for SA and *PR3*, *PR4*, and *LOX* as signature defence response genes for JA. Although there have been significant advances in the understanding of plant defences in model systems, signature defence genes associated with SA and JA in woody plants such as *Eucalyptus* has not been extensively explored.

*Eucalyptus* species and hybrid clones are commercially planted because of their valuable wood and fiber properties which have been exploited by the pulp and paper industry. Due to the importance and value associated with this genus of hardwood trees, the initiative to sequence the genome of *Eucalyptus grandis* was undertaken by the US Department of Energy (DOE—Joint Genome Institute) in 2008. Currently, the first annotated version of the genome, released in January 2011, is available through Phytozome v7.0 and consists of 4952 scaffolds including 11 linkage groups/chromosomal assemblies (Phytozome, 2010). This resource provides a useful platform for elucidating various physiological aspects of *Eucalyptus*, such as their responses to biotic and abiotic factors. Although *Eucalyptus* trees are generally disease tolerant, they can and do succumb to diseases caused by a wide range of pathogens (Wingfield et al., 2008). A stepping stone for improving our understanding of *Eucalyptus* responses would be to identify genes associated with the SA and JA signaling pathways in these trees. The first aim of this study was to identify *Eucalyptus* orthologs of signature defence genes specific for the SA (*PR2* and *PR5*) and JA (*PR3*, *PR4*, and *LOX*) signaling pathways using sequence information from other plant species and the *E. grandis* genome sequence. Secondly we aimed to characterize the expression profiles of the putative orthologs using reverse transcriptase quantitative PCR (RT-qPCR). Transcript profiling that was conducted under mock induction of the signaling pathways revealed dose-dependent induction of the orthologous signature defence genes, as well as key time points for their expression. Furthermore, the orthologous genes were found to corroborate the antagonistic relationship observed between SA and JA in *Arabidopsis*. The ability of these putative signature defence genes to respond to fungal infection by *Chrysoporthe austroafricana* was examined in tolerant (TAG5) and susceptible (ZG14) *E. grandis* genotypes (Van Heerden et al., 2005). Expression profiling of these signature genes revealed the possible involvement of SA in defence against *C. austroafricana*.

## Materials and methods

### Plant material

Disease free *E. grandis* (Clone A, Mondi Tree Improvement Research) plantlets were propagated *in vitro* and following rooting the plantlets were transferred to Jiffy pots and grown at 25–28°C under long day (16 h) conditions under light intensity of 300–500 lum/sqf. Potted cuttings of *E. grandis* clonal genotypes, ZG14 and TAG5 (Mondi) with a stem diameter of 1 cm, were subsequently used for the infection trial with *C. austroafricana* and kept under the same conditions as stated above.

### Phylogenetic identification of putative orthologs for signature defence genes associated with SA and MeJA

The *Arabidopsis thaliana* amino acid sequences of the genes of interest were obtained from The *Arabidopsis* Information Resource (TAIR, version 10) (https://www.arabidopsis.org). A BLASTP similarity search was conducted against the predicted *E. grandis* proteome (first *ab initio* and homology-based annotation) using the amino acid sequence as a query. This analysis was performed in Phytozome v7.0 (www.phytozome.net) and predicted *E. grandis* transcripts with *e*-values <10^−50^ were downloaded. Putative *Populus trichocarpa* orthologs of the gene of interest were retrieved from NCBI and added to the analysis using the same BLAST parameters. Aligned sequences were imported into MEGA v5.01 (Tamura et al., 2011) for the construction of a neighbor joining (NJ) tree. Confidence in the clades was substantiated by a bootstrap value calculated after 10,000 permutations. For the maximum likelihood (ML) analysis, the aligned sequences were assessed using Prottest 3.0 (Abascal et al., 2005) and PhyML 3.0 (Guindon and Gascuel, 2003) was used to perform the ML analysis using the parameters of the best model obtained from the Prottest results. Confidence in the clades was substantiated by a bootstrap value calculated after 1000 permutations. Furthermore the expression pattern of the selected gene model across different tissues was assessed on the *Eucalyptus* Genome Integrative Explorer (EucGenIE, http://eucgenie.bi.up.ac.za, Mizrachi et al., 2010). Following the identification of putative orthologs in *E. grandis* based on the expression data and NJ and ML trees, primers were designed and verified in Phytozome v7.0 using a BLASTN similarity search against the *E. grandis* genome (Table 1). *Eucalyptus* orthologs for *PR1a* (AT2G14610), *VSP1* (AT5G24780), and *PDF1.2* (AT5G44420) could not be identified based on the phylogenetic approach and were thus not assessed further.

**Table 1:** **Primer sequence of *Eucalyptus* target signature defence genes and reference genes assessed using RT-qPCR**.

**Primer name**	**Forward primer (5′–3′)**	**Reverse primer (5′–3′)**	**Amplicon size (bp)**
**SA SIGNATURE DEFENCE GENES**			
^*^EgrPR2	GCTCTACAACCAGCGCAATATC	GCCAACTGCTATGTACCTGAAC	214
*EgrPR5*	CCTGTTGGACGTCAACGCC	GTCGTCGTACTCGAAGATT	167
**JA SIGNATURE DEFENCE GENES**			
*EgrPR3*	CGGCCGCGAAGTCGTTCCC	AACTATAACTACGGGCAAT	277
*EgrPR4*	ATGCCGTGAGCGCATACTG	GCGTGTTGGTCCTGGTGTT	156
*EgrLOX2*	ATGAACACTTGCTTCCATT	TCCTACCATACGTGAACAA	165
**REFERENCE GENES**			
*EgrARF*	TGCGTACCGAGTTGTTGAGG	GTTGCACAGGTGCTCTGGAT	195
*EgrFBA*	TGAAGACATGGCAAGGAAGG	GTACCGAAGTTGCTCCGAAT	190
*EgrIDH*	TGGAACTGTTGAGTCTGG	TTAGGACCATGAATGAGGAG	59

* Egr, E. grandis.

### Dose response of putative orthologous signature defence genes for SA and MeJA signature defence genes

SA and MeJA phytohormones were administered to *E. grandis* (clone A) plantlets by spraying the aerial portions with varying concentrations of the inducers until run-off. The following inducer concentrations were assessed: 25 μM, 50 μM, 100 μM, 250 μM, 500 μM, 1 mM, and 5 mM. Sodium salicylate (Riedel-de Haen, Seelze, Germany) was used to prepare the SA solutions (adjusted to pH 7.0 with NaOH solution) with the addition of 0.1% Tween® 20 (Sigma-Aldrich, Missouri, USA). MeJA (methyl jasmonate 95%, Sigma-Aldrich) was prepared with the addition of 0.1% ethanol (100%) as well as 0.1% Tween® 20 (Sigma-Aldrich). Control plants for SA treatment were sprayed with distilled water containing 0.1% Tween® 20. The control plants for the MeJA treatment were sprayed with distilled water containing 0.1% Tween® 20 and 0.1% ethanol. Aerial parts of the plantlets were harvested 24 h post-treatment (hpt). Three biological replicates of consisting of five plants each was harvested for the control and treated samples.

### Investigation of the expression profiles of putative *E. grandis* orthologs over a time course

Phytohormones, SA and MeJA, were administered to *E. grandis* (clone A) plantlets as described in the previous section. A single concentration selected from the dose response experiment for SA and MeJA was assessed at the following time points: 6, 12, 24, and 48 hpt. Controls were harvested at each individual time point as well as at time zero which refers to the time prior to the application of inducers. Three biological replicates consisting of five plants each was harvested for the control and treated samples at the different time points.

### Infection trial with *chrysoporthe austroafricana*

Ramets of two *E. grandis* clones, TAG5 and ZG14 trees, with an approximate stem diameter of 1 cm were inoculated with the fungus *C. austroafricana* CMW2113 as previously described (Roux et al., 2003). Lesion lengths were recorded and plant material (stem tissue, 1 cm above and below the lesion) was harvested at 48 h post-inoculation, the earliest time point at which confirmation of infection was observed, as well as 2 and 6 weeks post-inoculation (wpi). Three biological replicates consisting of three trees each was harvested for the control and inoculated samples. Re-isolation of the fungus was performed by excising a piece from the periphery of the lesion after 6 weeks and placing the block on 2% Malt Extract Agar (Merck, Gauteng, South Africa). Confirmation of infection by *C. austroafricana* was done by observing the culture morphology after 5 days.

### RNA extraction and first strand cDNA synthesis

Total RNA was extracted from the plant powder using a modified cetyl-trimethyl-ammonium-bromide (CTAB) extraction protocol (Zeng and Yang, 2002). Extracted samples were treated with RNase-free DNaseI enzyme (Qiagen Inc, Valencia, CA) and subsequently column purified using the RNeasy® MinElute Kit (Qiagen Inc) as per the manufacturer's instructions. Purified RNA (1 μg) was used as the template for reverse transcription using Improm II reverse transcriptase enzyme (Promega, Wisconsin, USA).

### Reverse transcriptase quantitative PCR (RT-qPCR) analysis

Reverse transcriptase quantitative PCR was performed according to the Minimum Information for Publication of Quantitative Real-Time PCR Experiments guidelines (MIQE) (Bustin et al., 2009). For each target, three biological replicates and three technical replicates per biological replicate was performed. The LightCycler® 480 SYBR Green I Master Mix (2× concentration) kit (Roche, Mannheim, Germany) was used to perform the RT-qPCR experiments on the LightCycler® 480 Real-Time PCR system (Roche Diagnostics, GmBH, Basa, Switzerland) according to the manufactures instructions. Reactions were set up in 11 μl volumes containing: 1 μl (1:10 diluted cDNA template), 5 μl LightCycler® 480 SYBR Green I Master Mix, 0.5 μM of each primer, and water to make up the total volume. For each primer pair, a negative no template control was included. Samples were normalized to a combination of the following reference genes: *ADP ribosylation factor (EgrARF)*, *Fructose bisphosphate aldolase (EgrFBA)*, and *NADP-isocitrate dehydrogenase (EgrIDH*, Boava et al., 2010). Relative quantification and normalization was performed using *qBASE*plus v1.0 (Hellemans et al., 2007). The datasets were tested for normality using the Shapiro–Wilk's test with the statistical software package Analyse-it® (Analyse-it Software, Ltd., Leeds, UK). The pairwise comparison Kruskal–Wallis test (*p* < 0.05) was applied to investigate significant differential expression unless otherwise stated.

## Results

### Phylogenetic identification of putative orthologs for PR genes associated with the salicylic acid and jasmonic acid signaling pathways in *Eucalyptus grandis*

Putative orthologs of defence genes that are known to be responsive to the SA and JA signaling pathways from *Arabidopsis* were identified in *E. grandis* using BLAST algorithms and phylogenetic analyses (Table 2). All of the genes, except for *EgrPR2*, had predicted transcripts that were congruent with the annotated sequence of *E. grandis* located on Phytozome v7.0. Further investigation into *EgrPR2* revealed a region on scaffold 1:33791675_33792649 that had the highest similarity to the *Arabidopsis* candidate. Therefore an *ab initio* prediction of this region was performed using GeneMark (designated GM_*Egrandis*_V1_Scaffold1) and the result of this was included in the phylogenetic tree. The *Arabidopsis PR2* gene formed a clade with GM_*Egrandis*_V1_Scaffold1 that was accompanied by a strong bootstrap statistical support in the ML phylogenetic tree (Results not shown) and the GeneMark predicted gene model therefore was selected as the putative ortholog (Table 2).

**Table 2:** **Predicted gene models and corresponding genomic scaffold regions selected as putative orthologs for the SA and MeJA defence signature genes in *E. grandis***.

**Gene**	**TAIR ID**	**Predicted gene model**	**Genomic scaffold region**
*EgrPR2*	AT3G57260	^*^GM_*Egrandis*_V1_Scaffold1	Scaffold_1: 33791675–33792649
*EgrPR3*	AT3G12500	*Eucgr.I01495*	Scaffold_9: 25149898–25151718
*EgrPR4*	AT3G04720	*Eucgr.B02124*	Scaffold_2: 42319519–42320281
*EgrPR5*	AT1G75040	*Eucgr.A00487*	Scaffold_1: 7623283–7624480
*EgrLOX2*	AT3G45140	*Eucgr.J00825*	Scaffold_10: 8809509–8814780

* No predicted transcript on Phytozome v7.0 for the selected scaffold region.

### Expression profiling of the putative orthologous *EgrPR* genes at various concentrations of SA and JA reveals dose-specific induction

**Figure 1:**
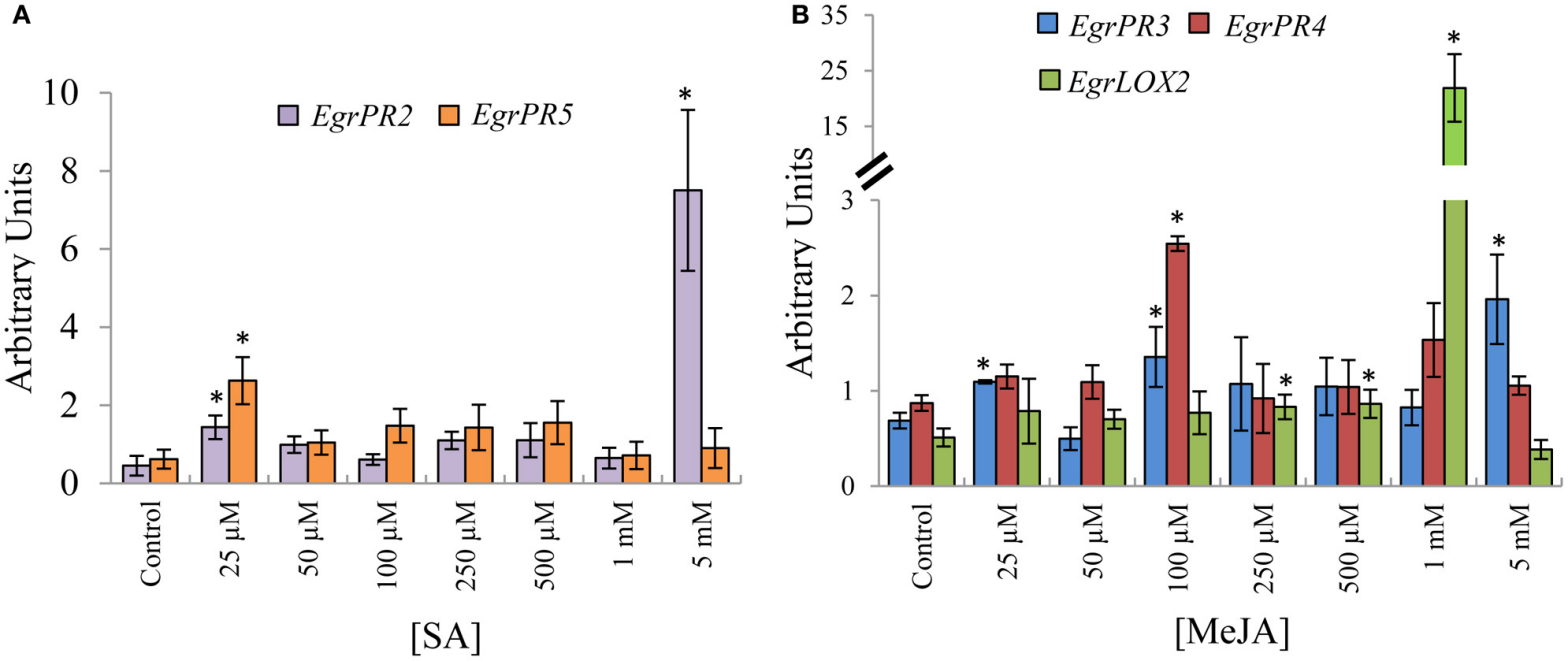
**Relative transcript abundance of *EgrPR* signature defence genes under hormone treatment. (A)** Putative SA signature defence genes following normalization with *EgrARF* and *EgrFBA*. **(B)** Putative MeJA signature defence genes following normalization with *EgrARF* and *EgrIDH*. The y-axis represents the relative transcript abundance ratios expressed in arbitrary units. The x-axis shows the respective concentration range that was applied to the aerial parts of *E. grandis* plants. Error bars show the standard error of the mean of the biological replicates (*n* = 3) sampled after 24 hpt. Significance, indicated by ^*^, is relative to the control for each target and was calculated by the Kruskal–Wallis test (*p* < 0.05).

Following the identification of putative orthologs of signature defence genes of the SA and JA signaling pathways, we investigated the expression profile of the candidates under various doses of phytohormone application. Putative orthologous defence signature genes for the SA pathway, *EgrPR2* and *EgrPR5* both displayed increased transcript abundance at 25 μM SA (Figure 1A). Although both targets had increased transcript abundance at 25 μM, *EgrPR2* had a much higher increase (16-fold compared to the control) at 5 mM and therefore this concentration was used for further experiments. Putative orthologs for the following candidates, *EgrPR3*, *EgrPR4*, and *EgrLOX2* were profiled as signature defence genes of the JA pathway. *EgrPR3* and *EgrPR4* exhibited increased transcript abundance at a common concentration of 100 μM. *EgrPR3* was also significantly increased at 25 μM and 5 mM but the fold change was lower than at 100 μM for *EgrPR4* (Figure 1B). Although the expression of *EgrLOX2* was significantly induced at 1 mM, it was decided to proceed with 100 μM for further experiments as both *EgrPR3* and *EgrPR4* exhibited significant differential expression at this concentration.

### Expression of *EgrPR* defence genes validate SA–JA antagonism in *E. grandis*

**Figure 2:**
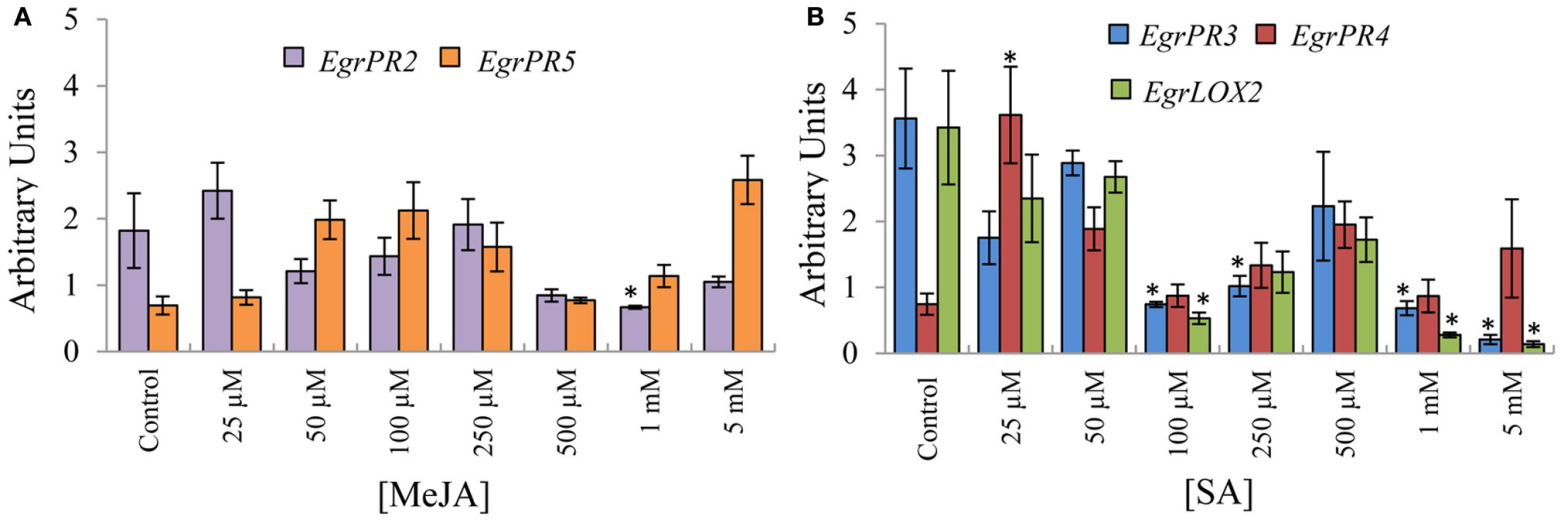
**Relative transcript abundance of the putative orthologs for the *EgrPR* defence genes in tissue treated with the opposite phytohormone.** The y-axis represents the relative expression ratios expressed in arbitrary units. Putative SA signature defence genes **(A)** were normalized with *EguIDH* and *EgrARF* whereas the putative MeJA signature defence genes **(B)** were normalized with *EgrARF* and *EgrFBA*. The x-axis represents the concentration range for the applied inducer. Error bars show the standard error of the mean of the biological replicates (*n* = 3). Significance, indicated by ^*^, is relative to the control in each graph and was calculated by the Kruskal–Wallis test (*p* < 0.05).

To investigate the hypothesis that SA and JA display an antagonistic relationship, the candidates were assessed by profiling the SA defence signature genes in material induced with MeJA and *vice versa*. The antagonistic relationship between SA and JA was clearly validated to occur in *E. grandis* in tissue treated with the phytohormone at selected concentrations. *EgrPR2* was suppressed at higher concentrations of MeJA relative to the control (Figure 2A). *EgrPR3* expression was reduced at 100 μM, 250 μM, 1 mM, and 5 mM whereas *EgrLOX2* was significantly lower at 100 μM, 1 mM, and 5 mM SA. *EgrPR4* had higher abundance at 25 μM SA and was not repressed at any of the other concentrations (Figure 2B).

### Time-dependent expression of putative *EgrPR* genes identifies key points of induction

**Figure 3:**
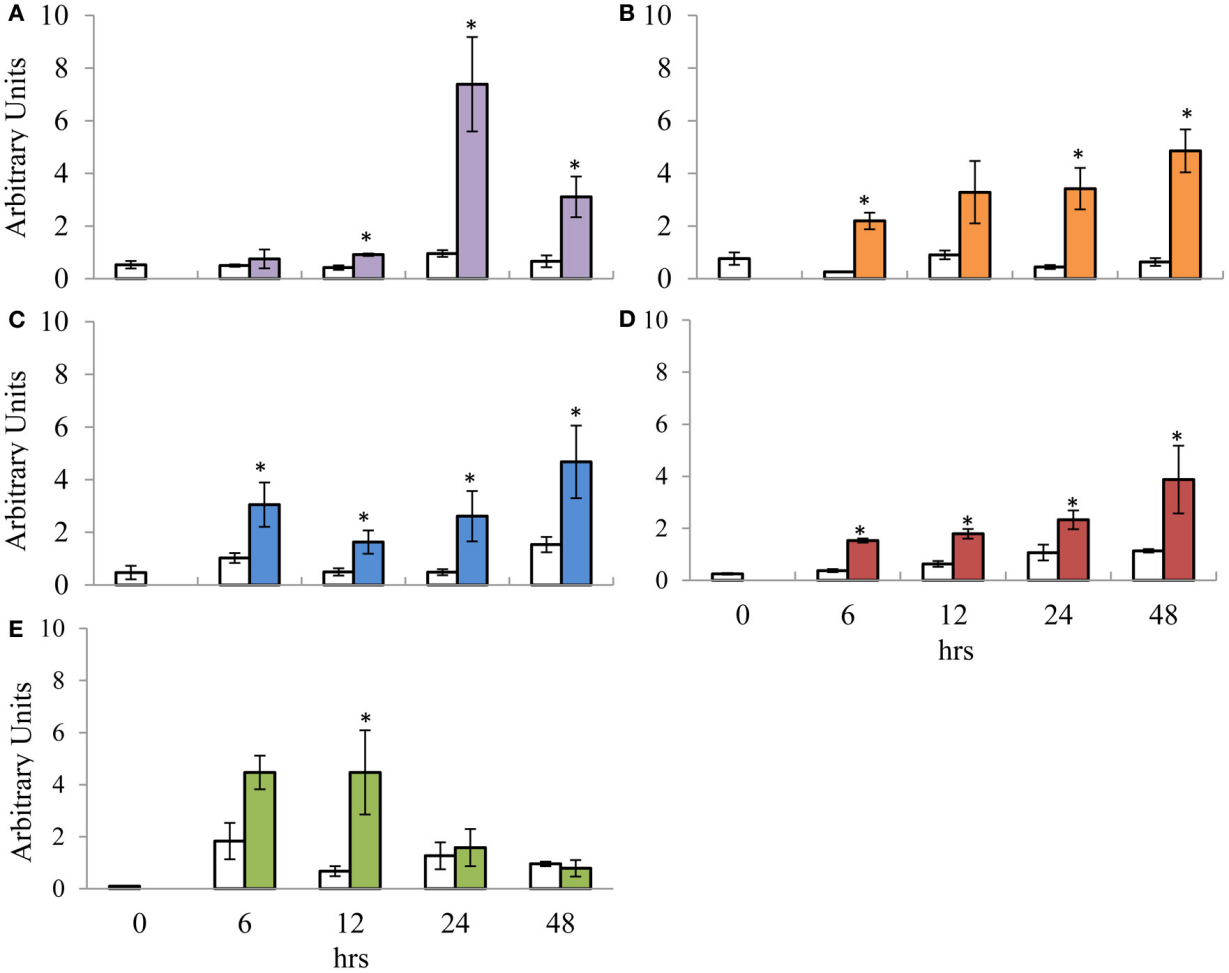
**Relative transcript abundance of putative orthologs for SA and MeJA signature defence genes assessed during the time course trial. (A)** *EgrPR2*; **(B)** *EgrPR5*; **(C)** *EgrPR3*; **(D)** *EgrPR4*; **(E)** *EgrLOX2*. The y-axis represents the relative expression ratios expressed in arbitrary units. The x-axis represents the time course (h) post-treatment with 5 mM SA **(A** and **B)** and 100 μM MeJA **(C–E)**. Samples were normalized with *EgrARF* and *EgrFBA*. Error bars are show the standard error of the mean of the biological replicates (*n* = 3). White boxes represent the control samples whereas the colored boxes represent the treated samples. Significance between the control and treated samples is indicated by ^*^ at a specific time point and was calculated by the Kruskal–Wallis test (*p* < 0.05).

To investigate the expression profile of the suite of signature defence genes over a time course, 100 μM MeJA and 5 mM SA was applied to aerial portions of the *E. grandis* (clone A) tissue culture plants and the harvested material was profiled over various time points. The relative expression values for each time point was compared to the *T* = 0 control as well as the time specific control using the Kruskal–Wallis test (Note that the significance indicated on the graphs is only in relation to the time specific control). The *T* = 0 control was included in the experiment to indicate the basal level of gene expression prior to any treatment. Transcript abundance of the SA signature defence gene candidate, *EgrPR2* was significantly increased at 12, 24, and 48 hpt with a drastic peak at 24 hpt followed by a decline at 48 hpt (Figure 3A). *EgrPR5* displayed a gradual increase in expression from 6 to 48 hpt, with the expression of the target showing statistical significance all the time points except 12 hpt (Figure 3B). Signature defence genes for JA, *EgrPR3*, *EgrPR4*, and *EgrLOX2* all displayed altered levels of expression at the various time points (Figures 3C–E). *EgrPR4* transcript levels increased progressively from 6 to 48 hpt, with all the time points being statistically significant (Figure 3D). Notably the level at which *EgrPR2* and *EgrPR4* are expressed at 24 hpt was approximately the same level as was observed in the dose response experiment, thereby indicating reproducibility of the results.

### Expression profiling of the putative orthologous defence genes during infection by a pathogen qualifies the potential of the candidates to be diagnostic of SA and MeJA and implicates SA in defence against *C. austroafricana*

**Figure 4:**
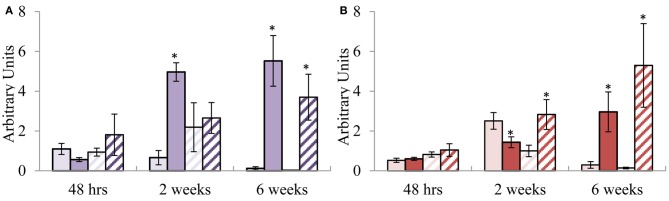
**Relative transcript abundance of putative orthologs for *EgrPR* signature defence genes during infection with *C. austroafricana.* (A)** *EgrPR2*; **(B)** *EgrPR4*. The y-axis represents the relative expression ratios expressed in arbitrary units. The x-axis represents the time points post-inoculation at which the samples were analyzed. Error bars show the standard error of the mean of the biological replicates (*n* = 3). Samples were normalized to *EgrARF* and *EgrFBA*. Light and dark solid boxes represent the TAG5 control and inoculated samples respectively whereas the light and dark striped boxes represent the ZG14 control and inoculated samples respectively. Significance, indicated by ^*^, is relative to the control and was calculated by the Student's *t*-test (*p* < 0.05).

The potential of these defence signature genes to be used as diagnostic markers was investigated under pathogen stress by employing the *E. grandis*—*C. austroafricana* pathosystem. Using the Kruskal–Wallis statistic test a significant difference (*p* = 0.0295) was observed between the lesion lengths of TAG5 (4.8 ± 2.1 cm) and ZG14 (8.2 ± 3 cm) at 6 wpi whereas no significance was observed at 48 h and 2 wpi. In TAG5, the SA signature gene *EgrPR2* showed significant differential expression at 2 and 6 wkpi (Figure 4A). In TAG5, the JA signature genes, *EgrPR4* significantly decreased at 2 wpi and increased once again at 6 wpi. Despite significant up-regulation of *EgrPR4* at 6 wpi in TAG5 compared to its control, the level to which it was induced was lower than *EgrPR2* levels (Figure 4B). In ZG14, the level of expression of *EgrPR2* was only significantly up-regulated at 6 wpi (Figure 4A) whereas the expression of *EgrPR4* transcripts was found to be significantly up-regulated at 2 and 6 wpi (Figure 4B). The pre-treatment of the susceptible genotype of *Eucalytpus* with 5 mM SA, prior to manual inoculation with *C. austroafricana*, resulted in a smaller lesion lengths (5 ± 0.5 cm) compared to the untreated plants (7 ± 0.6 cm) at 5 wpi (One-Way ANOVA, *p* < 0.05). These lesion lengths were comparable to lesions found on the tolerant genotype (4.8 ± 0.4 cm).

## Discussion

*PR* genes have been shown to be indicators of the SA and MeJA signaling pathways and can be termed signatures of these pathways. This study aimed to identify orthologs of signature defence genes for the SA and MeJA signaling pathways from *A. thaliana* in *E. grandis* using sequence similarity and phylogenetic analysis. Phylogenetics provides a solid starting point for selecting candidates to investigate, however it does not provide definitive evidence that the selected gene is the true functional ortholog (Chen et al., 2007). *Eucalyptus*, *Populus*, and *Arabidopsis* share an ancient hexaploidization event and therefore on average there should be three genes in each species relative to the ancestor (Jaillon et al., 2007). These genes may have undergone various gene loss and/or duplication events which have changed this number for many genes and gene families thereby possibly creating multiple functional orthologs. The putative orthologous signature defence genes identified here provide suitable candidates for further investigation in complementation and functional studies to better understand the role of these genes in *E. grandis*.

### Orthologs for *EgrPR* signature defence genes exhibit dose-specific induction and pathway specificity for *EgrPR2*, *EgrPR4*, and *EgrLOX*

Based on the premise that the candidates identified through phylogeny were defence signature genes for SA and JA, we subsequently investigated the expression of these targets under various doses of phytohormone treatment. The concentrations used in this study were based on experiments conducted in *A. thaliana* and on the level of the phytohormone following a pathogen challenge in other model organisms (Rasmussen et al., 1991; Jung et al., 2007). The transcript abundance levels of the putative SA signature defence genes, *EgrPR2* and *EgrPR5* were increased (Figure 1A) by application of the inducer which was consistent with literature in *Arabidopsis* (Reymond and Farmer, 1998; Kunkel and Brooks, 2002; Delaure et al., 2008). Furthermore, when these genes were evaluated for their specificity to the SA pathway, it was clearly demonstrated that expression levels of *EgrPR2* was suppressed by higher concentrations of MeJA (Figure 2A). These results suggest that in *E. grandis EgrPR2* could serve as a diagnostic signature gene for the SA pathway. Expression of the MeJA defence signature genes, *EgrPR3* and *EgrLOX2* were significantly differentially regulated at varying concentrations of this phytohormone. These signature genes were additionally repressed at high concentrations of SA treatment confirming the suppressive effect of SA on MeJA responses. Transcripts of *EgrPR4* were found to be up-regulated by the application of MeJA, but showed no differential expression under SA treatment other than at 25 μM. Nonetheless, *EgrPR4* can be utilized as a defence signature for MeJA as expression levels of this gene were significantly altered upon application of that phytohormone. The data suggests that the known antagonistic relationship between MeJA and SA in *Arabidopsis* may also occur in *Eucalyptus*. All of the MeJA responsive defence signature genes profiled in this study were found to be diagnostic to the MeJA pathway in *E. grandis* and could serve as suitable markers for the pathway. Although SA and JA predominantly have an antagonistic relationship (Pieterse et al., 2009), there have been situations whereby these pathways act synergistically (Mur et al., 2006; Lazniewska et al., 2010). The outcome of the interaction between SA and JA seems to be largely dependent of the timing of activation and the concentration of the phytohormones (Mur et al., 2006).

### Time dependent expression profiles suggest that MeJA and SA signature defence genes in *E. grandis* are differentially regulated as early as 6 hpt

To further elucidate the expression profiles of the *Eucalyptus* signature defence genes, we investigated the response of the candidates over time. The time at which a host's defences are activated has a crucial role in determining the outcome of a pathogen interaction. Susceptibility may not only be due to the lack of required artillery (e.g., defence genes), but also to the delayed activation of the genes required to curb the pathogen (Loon, 2009). Elucidating the time dependent expression profiles of the putative orthologous signature genes under mock induction of the signaling pathways would provide a glimpse into how the genes would respond under pathogen conditions. SA hormone levels in tobacco plants infected with tobacco mosaic virus (Malamy et al., 1990, 1996) parallels the expression profile observed for *EgrPR2* in this study (Figure 3A) under application of SA in *E. grandis*. In contrast to *EgrPR2*, *EgrPR5* was shown to gradually increase over the time points with a maximum expression level detected at 48 hpt (Figure 3B). This suggests that the signature defence genes identified in this study respond to mock induction of the signaling pathway in a similar manner as they would under pathogen incursion. Tobacco plants that have been treated with exogenous MeJA displayed time course patterns similar to that found in *E. grandis* for *LOX* and *PR3* (Bell and Mullet, 1993). In *E. grandis*, *EgrLOX2* transcript levels were significantly up-regulated as early as 12 hpt followed by a decline at 24 hpt (Figure 3E). This could indicate a possible role for *EgrLOX2* in the early stages of defence activation in a host as this gene is involved in jasmonate biosynthesis. *EgrPR3* displayed a similar profile with the level of transcripts increasing from 6 to 48 hpt in *E. grandis* (Figure 3C) compared to increasing levels from 8 to 24 h post-MeJA treatment in tobacco (Rickauer et al., 1997). A microarray time course study in which *Arabidopsis* plants were treated with MeJA revealed that *EgrPR4* transcripts began to increase as early as 1 h then slowly declined by 24 h (Jung et al., 2007). Conversely in *E. grandis*, *EgrPR4* increased from 6 hpt with the maximum expression level detected at 48 hpt (Figure 3D). Although the time points differ between the two organisms, the general trend of expression remains the same. The observed increase in the transcript levels of *EgrPR3* and *EgrPR4* over time could also be due to the role of these proteins in the host during defence. Both of these genes encode for products that target and alter the cell wall composition of a fungal pathogen and during infection an increase in expression would be beneficial in preventing the spread of the pathogen (Selitrennikoff, 2001).

### Pathogenicity experiments conducted with *C. austroafricana* establishes the diagnostic potential of the *EgrPR* signature defence genes and elucidates the importance of SA in defence against this pathogen

In *Arabidopsis*, the involvement of a specific signaling pathway during an interaction with a pathogen can be elucidated by the diagnostic ability of the assigned signature genes. This study examined the diagnostic potential of the putative orthologous signature genes for SA and MeJA found in *Eucalyptus* upon infection with *C. austroafricana.* It was found that at 2 wpi there was no substantial difference in lesion length between TAG5 and ZG14 whilst at 6 wpi there was a significant lesion difference, suggesting that during the initial 2 weeks following infection the tolerant host was able to initiate a certain response to curb the spread of the disease. Interestingly, the signature defence gene expression profiles that were observed in the two hosts suggest a probable role of SA in the tolerance mechanism of TAG5. In the incompatible interaction (TAG5 and *C. austroafricana*), at 2 and 6 wpi, *EgrPR2* transcripts were considerably up-regulated compared to the control, whereas up-regulation only occurred at 6 wpi in the compatible interaction (ZG14 and *C. austroafricana*) and to a lower lever (Figure 4A). In addition, the level of MeJA signaling at 2 wpi was lower in the incompatible interaction compared to the compatible interaction as indicated by the expression levels of *EgrPR4* (Figure 4B). The antagonistic relationship between SA and MeJA evidently occurs within these hosts at this time point and could possibly have a key role in determining the outcome of the interaction with *C. austroafricana*. From other plant species, *PR2* is known to encode for the β-1, 3-glucanase enzyme which facilitates the enzymatic degradation of the glucan component of fungal cell walls (Theis and Stah, 2004). In TAG5, the elevated level of *EgrPR2* could contribute to confining the spread of *C. austroafricana* by hydrolyzing the β-1, 3-glucan component of the cell wall. In the review by Selitrennikoff (2001), it's hypothesized that this particular glucan component maybe abundant in the hypal apex of a growing fungus and degradation of the β-1, 3-glucan may lead to a loss in rigidity of the cell wall thereby resulting in cell lysis and eventual cell death. *EgrPR2* was significantly up-regulated at the later time points (2 and 6 wpi) but not at the early time point of 48 hpi (Figure 4A) suggesting that the lack of an early response could be a partial reason as to why TAG5 is tolerant but not fully resistant against *C. austroafricana*. Based on the premise that SA may facilitate tolerance, ZG14 plants were sprayed with 5 mM SA to determine if this hormone would increase the tolerance of this host. A significant reduction in the lesion lengths of ZG14 treated with SA was observed and the lesions were of similar length to that seen in the tolerant TAG5 plants. Induction of systemic resistance in *E. urophylla* upon application of 5 mM SA has been previously documented (Ran et al., 2005). *EgrPR4* encodes a hevein-like protein which acts like a chitin binding protein by targeting the β-chitin component of the cell wall. These proteins migrate to the cell walls of an invading fungus and disrupt the formation of the septa and hyphal tips (Selitrennikoff, 2001; Theis and Stah, 2004). In ZG14, *EgrPR4* was elevated at 2 wpi however the host was still susceptible to *C. austroafricana*. A possible explanation for this is that the level to which this gene is expressed was not high enough to curb the pathogen. Timing of defence gene expression is crucial in a pathogen interaction and the lack of significant *EgrPR4* expression at 48 h in TAG5 or in ZG14, may contribute to the ability of *C. austroafricana* to proliferate within these hosts during the initial 2 weeks of infection.

Our results suggest that *EgrPR2* and *EgrPR4* were diagnostic of SA and MeJA signaling pathways respectively against *C. austroafricana* as SA was recognized as playing a role in enhancing tolerance against the pathogen in *Eucalyptus*. It is possible that other signaling pathways may have a role in contributing to resistance in this interaction. The involvement of SA in facilitating a defence response to a necrotrophic pathogen is in contrast to the published literature from *Arabidopsis* which implicates the involvement of the MeJA pathway (Glazebrook, 2005). In spite of this, there have been studies that have shown that SA could also assist in impeding necrotrophic pathogens (Ferrari et al., 2003; Azaiez et al., 2009). It may also be possible that in tree species the roles of SA and MeJA in pathogen defence could differ from what is known in *Arabidopsis.*

This study provides a first step toward understanding hormone mediated defence responses of *Eucalyptus* trees. It is envisaged that expression profiling of the diagnostic markers, *EgrPR2* and *EgrPR4*, can be adopted as a tool to determine which of the two major defence pathways are active against different pathogens in *Eucalyptus* in future.

### Conflict of interest statement

The authors declare that the research was conducted in the absence of any commercial or financial relationships that could be construed as a potential conflict of interest.
